# miR-548c-5p inhibits proliferation and migration and promotes apoptosis in CD90^+^ HepG2 cells

**DOI:** 10.2478/v10019-012-0025-z

**Published:** 2012-04-11

**Authors:** Lin Fang, Hai-Bing Zhang, Hua Li, Yong Fu, Guang-Shun Yang

**Affiliations:** 1 Department of Laboratory Centre, Shanghai Tenth People’s Hospital, Tongji University School of Medicine, Shanghai. China; 2 5th Department of Liver Surgery, Eastern Hepatobiliary Surgery Hospital, Second Military Medical University, Shanghai, China

**Keywords:** liver cancer stem cells, miR-548c-5p, apoptosis, NF-κB, *β-catenin*

## Abstract

**Background:**

Since the introduction of the theory of tumour stem cells (TSCs), the liver cancer stem cell (LCSC)-like cells have become one of the focuses in the research on liver cancer.

**Materials and methods.:**

In this study, CD90^+^ cells were applied as the possible LCSC-like cells, and the miRNA and gene expression were analyzed in the CD90^+^ HepG2 cells. The pilot study showed miR-548c-5p exerted potential effect on the CD90^+^ HepG2 cells and was thereafter applied for the further study. CD90^+^ HepG2 cells were assigned to miR-548c-5p mimic transfection group and control group. MTT assay was performed to detect the proliferation of CD90^+^ HepG2 cells. The migration and invasion abilities were examined by wound healing assay and transwell migration assay, respectively. A detection of apoptosis was performed by fluorescence microscopy.

**Results:**

Our results showed that *caspase-3* and *bcl-2* were down-regulated while *caspase-8* was up-regulated in the CD90^+^ HepG2 cells. Moreover, the miR-548c-5p transfection could down-regulate the expression of *β-catenin, Tg737, bcl-2, bcl-XL*, and *caspase-3*, inhibit the proliferation, migration and invasion and promote the apoptosis of the CD90^+^ HepG2 cells.

**Conclusions:**

Our findings indicate the imbalance between apoptosis and anti-apoptosis in the LCSC-like cells, which influence the biological features of LCSC-like cells. miRNA plays a regulatory role in the LCSC-like cells among which miR-548c-5p might be a suppressor.

## Introduction

The pathogenesis of primary liver cancer remains unclear. Since the introduction of the theory of tumour stem cells (TSCs), the liver cancer stem cells (LCSC) have become one of the focuses in the research on liver cancer. The theory of LCSC concerns the tumour formation and biological features at the cellular level and the new explanations for the development and progression of tumours, which provide a new direction for the future research on liver cancer.^1^–^5^ However, there is no report on the existence of LCSCs in tumour tissues.

There is a difficulty in the isolation and biological investigations of LCSCs, due to the absence of specific marker for LCSCs. Studies have revealed that CD90 could be used as a possible surface marker of LCSCs.^6^–^8^ When CD90^+^ cells isolated from liver cancer cell lines and liver tissues of liver cancer patients were inoculated into severe combined immunodeficiency (SCID) rats, animals developed cancer showing that these cells were carcinogenic.^6^ The diagnosis of primary liver cancer was also confirmed by pathological evaluation. CD90^+^ cells were also detected in liver cancer patients^7^, suggesting that CD90 could be used as a valuable surface marker of LCSCs, and as the target of gene diagnosis and therapy. CD133 has been also considered a specific surface marker of LCSCs, because CD133^+^ cells have biological features of LCSCs.^9^–^11^ Side population (SP) cells refer to normal stem cells whose membrane contains ATP-binding cassette transporter, which can expel Hoechst33342 dye from the inside to the outside of the membrane effectively, while these characteristics are absent in the non-SP cells. It has been demonstrated that 100 SP cells isolated from liver cancer cells were enough for carcinogenesis, while non-SP cells had no carcinogenic ability, and SP could be used as a method to isolate LCSC-like cells.^12^ However, LCSC-like cells isolated by the above method are by no mean true LCSCs. In the present study, CD90^+^ cells were used as the possible LCSC-like cells for the further study.

The expression of multiple tumour suppressor genes is abnormal in the liver cancer, of which *p53* and *Tg737* are believed to be closely associated with the occurrence of liver cancer by affecting the LCSC-like cells.^13^,^14^ The Wnt and NF-κB signalling pathways play important roles in regulating the LCSC-like cells.^15^,^16^ Apoptosis plays an essential role in not only the growth and development of cancer cells but in various diseases including tumours, immune diseases, infectious diseases and neurological diseases. Anti-apoptosis is an important process in the development and progression of tumours, and apoptosis also exists in liver cancer cells.^17^,^18^

miRNAs are a group of endogenous non-protein-encoding single-stranded low-molecular-weight RNA with a length of about 20–25 nt, widely existing in the eukaryotes. miRNAs play important roles in the cell proliferation, differentiation and apoptosis.^19^ However, the specific role of miRNAs in the LCSC-like cells is still poorly understood.

In the present study, CD90 was used as a possible marker for LCSC-like cells, and these LCSC-like cells were isolated from liver cancer cells HepG2, and changes in the expression of miRNAs, cancer-inhibiting genes and apoptosis-related genes were determined. Our results showed the difference in expressions of miR-548c-5p, miR-145, miR-375, miR-874, miR-155, miR-198 and miR-1289 between CD90^+^ HepG2 cells and CD90^−^ HepG2 cells, of which our pilot study revealed miR-548c-5p could affect the proliferation and promote the apoptosis of the CD90^+^ HepG2 cells. Thus, miR-548c-5p was further studied, hoping to gain a preliminary understanding of the effect of miR-548c-5p on the CD90^+^ LCSC-like cells and provide evidence for the understanding of biological features of LCSC-like cells.

## Materials and methods

### Cell line and culture

Human liver cancer cell line HepG2 (Shanghai Institutes for Biological Sciences of the Chinese Academy of Sciences, China) was maintained in monolayer cultures in high glucose Dulbecco’s Modified Eagle Medium (DMEM; Gibco, USA) containing 10% (v/v) foetal bovine serum (FBS; Gibco, USA) and 1% (v/v) penicillin (Weihui Biotechnology Co. Ltd., China) at 37°C in a humidified atmosphere of 5% CO_2_. Cells in the exponential growth phase were harvested and a cell suspension was prepared at a density of 3.0×10^4^ cells/mL and added into 96-well plates for transfection.

### Screening of CD90^+^ HepG2 cells

After the addition of PE-labelled anti-human CD90^+^ monoclonal antibody (BD Biosciences, USA), the cells were mixed with EasySep® PE Selection Cocktail and cultured by addition of EasySep® magnetic beads (Baitong Co., Ltd, China). The CD90^+^ cells were isolated from the HepG2 cells by magnetic activated cell sorting (MACS; Baitong Co., Ltd, China).

### Detection of gene and miRNA expression by real-time-PCR assay

Total RNA was extracted from the CD90^+^ HepG2 cells with TRIzol (Invitrogen, USA) and cDNA was synthesized from 2 μg of total RNA using Moloney murine leukaemia virus (MMLV) reverse transcriptase (Promega, USA). Real-time PCR was performed on ABI 7300 Real-time PCR System (Applied Biosystems, USA). The expression of the related genes (*p65, β-catenin, p53, Tg737, bcl-2, bcl-XL, caspase-3* and *caspase-8*) was analyzed using SYBR ExScript real-time PCR Kit (Tiangen Biotech Co., Ltd., China). Briefly, 20 μL of reaction mix was prepared. Thermal cycle conditions were as follows: 95°C for 30 s; 40 cycles of 95°C for 5 s and 60°C for 32 s. The expression levels of target genes were determined by using the 2^−^^ΔΔCt^ method. GAPDH was used as an endogenous control. A detection was carried out in triplicates.

miRNA was extracted with TRIzol similar to total RNA isolation for miRNA reverse transcription and amplification followed by the detection of expressions of miR-155, miR-198, miR-373, miR-548c-5p, miR-1289, miR-145, miR-375, miR-874, miR-1183 and miR-1324 using SYBR chemistry (Tiangen Biotech Co., Ltd, China).

The expression of selected genes and miRNAs in CD90^+^ HepG2 cells was compared to those in CD90^−^ HepG2 cells. The relative expression of the mRNA was calculated with the following formula:

$$\begin{array}{l} \Delta \text{CT}=\text{CT}\;\text{sample}-\text{CT}\;\text{endogenous}\;\text{control}\\ \text{Relative}\;\text{expression}=2^{-\Delta \text{CT}(\text{CD}90+)}/2^{-\Delta \text{CT}(\text{CD}90-)}\times 100\% \end{array}$$

### Cell transfection and detection of selected genes

CD90^+^ HepG2 cells were assigned to miR-548c-5p group and control group. Cells in miR-548c-5p group were transfected with miR-548c-5p mimics (GenePharma. Co., Ltd, China) according to manufacturer’s instructions. Cells in the control group were exposed to neither Lipofect transfection reagent nor miR-548c-5p mimics. miR-548c-5p mimics were mixed with Lipofect transfection reagent (Promega, Madison, WI, USA) followed by 10 min incubation. The concentration of miR-548c-5p mimics was diluted to 50 nmol/L before transfection. Cells were incubated with 100 μl of transfection mixture and collected 24 h and 48 h later for the use in further assays. All transfections were carried out in triplicates. miRNA was extracted at 24 h and 48 h after the transfection and the miR-548c-5p expression was detected by real time-PCR to verify the transfection efficiency. Total RNA was extracted from the transfected CD90^+^ HepG2 cells which highly express miR-548c-5p, and real time-PCR was employed to detect the mRNA expression of related genes (*p65, β-catenin, p53, Tg737, bcl-2, bcl-XL, caspase-3* and *caspase-8*).

### Caspase-3, bcl-2 and bcl-xl protein expression

Total protein was extracted at 48 h after the transfection with Total Protein Extraction Kit (BioChain, USA) according to manufacturer’s instructions. In brief, 100 μg of proteins in each group was subjected to 10% SDS/PAGE and transferred onto PVDF membranes (Millipore, USA). The membranes were subsequently incubated with rabbit anti-caspase-3, bcl-2 and bcl-xl antibodies (1:800; Santa Cruz Biotechnology, USA), and rabbit anti-β-actin (1:1,000; Santa Cruz Biotechnology, USA). After the incubation with horseradish peroxidase conjugated secondary anti-rabbit antibody (1:5,000–10,000; Santa Cruz Biotechnology, USA), visualization was performed with an enhanced chemiluminescence kit (Supersignal West Pico Chemiluminescent Substrate Kit, Pierce, USA) followed by exposure to X-ray film (Kodak, USA). β-actin served as an endogenous control. Experiment was performed three times.

### MTT assay

MTT assay was performed to detect the proliferation of CD90^+^ HepG2 cells. miR-548c-5p mimics were independently diluted to 20, 40, 60, 80 and 100 nmol for the transfection. The transfected cells were maintained in the 96-well plate at 37°C in humidified air with 5% CO_2_. In brief, MTT solution (5 mg/ml; Sigma, USA) was prepared with PBS and stored at 4°C. At 24, 48 and 72 h after the transfection, 20 μl of MTT was added to each well followed by incubation at 37°C for 5 h. After the addition of 150 μl of DMSO to each well to dissolve crystals, optical density (OD) was measured at 490 nm. The experiment was performed in quadruplicates. The inhibition rate (IR) of transfected CD90^+^ HepG2 cells was calculated as follows: IR = (1-OD treated / OD untreated) × 100%.

### Wound healing assay

In the *in vitro* wound healing assay, transfected CD90^+^ HepG2 cells were grown in 6-well plates until the cell confluence reached about 80%. Then, a scratch was made in each well using a sterile pipette tip, and cells were then maintained at 37°C in an atmosphere with 5% CO_2_. Wound healing was observed under a light microscope and images were captured at the same site at 0, 12, 24 and 48 h after scratching to observe the process of wound healing. The experiments were repeated twice and representative photographs are shown.

### Invasion assay

A transwell invasion assay was performed in miR-548c-5p mimics group and control group with CHEMICON Cell Invasion Assay Kit (Chemicon, USA). Transwell chambers were pre-coated with Matrigel. Cells were added to the Transwell system 48 h after the transfection. The lower chambers were filled with 500 μl of DMEM containing 15% FBS. The Transwell system was placed in the 24-well plate followed by the incubation for 48 h at 37°C in an atmosphere with 5% CO_2_. After removing the Matrigel and cells in the upper chambers, the membrane was stained with 0.1% crystal violet and observed under a microscope. Five fields were randomly selected from each membrane, and the number of cells penetrating the membrane was counted at a magnification of ×400. The invasion ability was expressed as the number of invading cells. The invasion of each group was assayed in triplicates. The experiment was repeated three times.

### Detection of apoptosis by fluorescence microscopy

CD90^+^ HepG2 cells were collected at 48 h after the transfection with miR-548c-5p by centrifugation at 300× g for 5 min at room temperature. Cells were washed once in cold PBS, and gently re-suspended in Annexin V Incubation Reagent (Trevigen, USA). Annexin V Incubation Reagent was prepared according to the manufacturer’s instructions. Cells were incubated in dark for 15 min at room temperature, then washed once in 1× Binding Buffer (Trevigen), and re-suspended in 100 μl of 1× Binding Buffer. Cell suspension was added onto cover slip and dried for a few minutes. Before the cell suspension completely dried, a drop of fluorescence mounting media (Trevigen, USA) was added onto the cover slip. Cells were observed under a fluorescence microscope (Olympus, Japan) at 540 nm to detect cells positive for propidium iodide and at 490 nm to detect cells positive for Annexin V-FITC.

### Statistical analysis

SPSS version 13.0 software program was employed for the statistical analysis. Data were expressed as mean ± standard error (SEM). A statistical analysis was performed by using one way analysis of variance followed by least significant difference (LSD) post-hoc test. A two-tailed Student’s paired t-test was also performed to compare the differences between 2 groups. A value of *P*<0.05 was considered statistically significant.

## Results

### miRNA expression in CD90^+^ HepG2 cells

**FIGURE 1:**
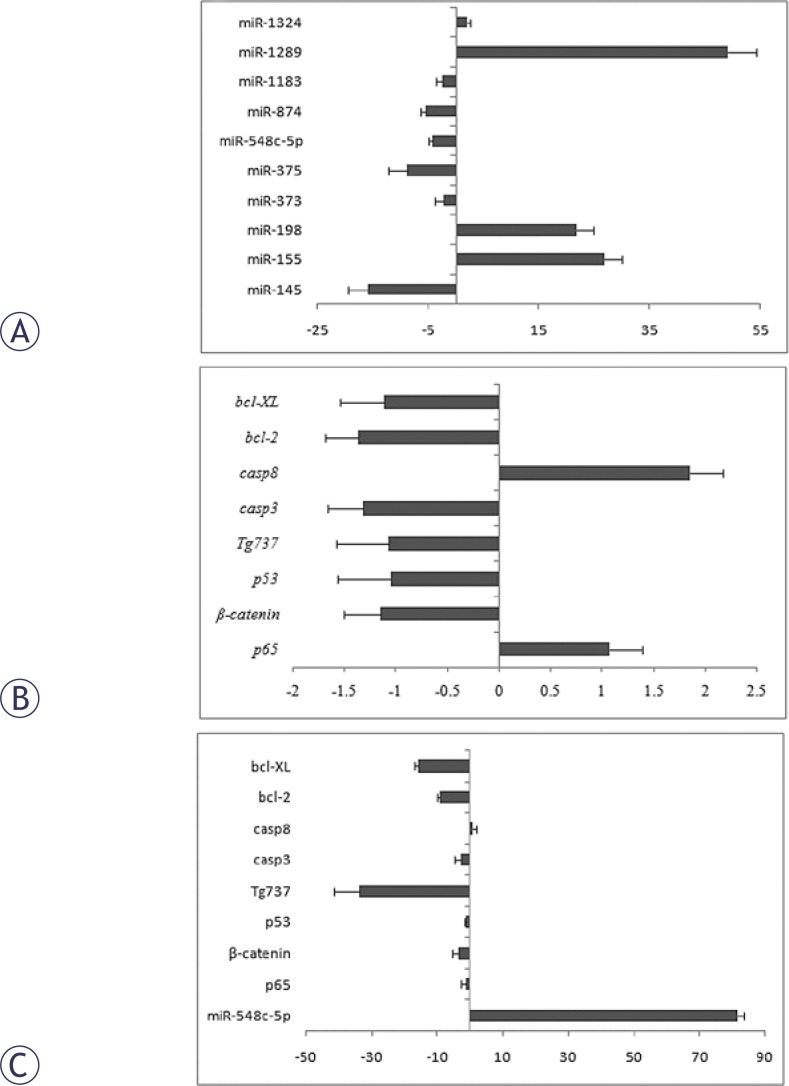
miRNA and gene expressions in CD90^+^ HepG2 cells as compared to CD90^−^ HepG2 cells. (A) Fold-change in miRNA expressions in CD90+ HepG2 cells. (B) Fold-change in expression of target genes in CD90^+^ HepG2 cells. (C) Fold-change in expression of target genes 48 h after miR-548c-5p transfection as compared to untransfected CD90^+^ HepG2 cells. Bars represent the mean value. Positive fold change represents up-regulation and negative fold change represents down-regulation.

Differences in miRNA expression in CD90+ HepG2 cells and in CD90− HepG2 cells were observed (Figures 1A). The expression of miR-548c-5p, miR-145, miR-375 and miR-874 decreased significantly in the CD90^+^ cells (P<0.05) when compared with CD90^−^ cells, while the expression of miR-155, miR-198 and miR-1289 were higher in the CD90^+^ cells than those in the CD90^−^ cells (P<0.05). However, there was no marked difference in the expression of miR-373, miR-1183 and miR-1324 between two groups.

### Expression of p65, β-catenin, p53, Tg737, bcl-2, bcl-XL, caspase-3 and caspase-8 in CD90^+^ cells

The expression of *caspase-3* and *bcl-2* was significantly down-regulated, and that of *caspase-8* markedly up-regulated in the CD90^+^ cells when compared with the CD90^−^ cells (both P<0.05). There was no significant difference in the expression of *p53*, *p65, β-catenin, Tg737* and *bcl-XL* (P>0.05) (Figures 1B).

### Expression of p65, β-catenin, p53, Tg737, bcl-2, bcl-XL, caspase-3 and caspase-8 in CD90^+^ cells following miR-548c-5p transfection

The miR-548c-5p expression in the CD90^+^ cells following transfection with miR-548c-5p mimics was increased by 80 fold as compared to untransfected cells (P<0.05). In the miR-548c-5p transfected CD90^+^ cells, the expression of *β-catenin, Tg737, bcl-2, bcl-XL* and *caspase-3* was down-regulated significantly (P<0.05) when compared with untransfected cells. There was no marked difference in the expression of *p65, p53* and *caspase-8* between CD90^+^ cells before and after the transfection (P>0.05) (Figures 1C).

### Protein expression of caspase-3, bcl-2 and bcl-xl in CD90^+^ cells following miR-548c-5p transfection

**FIGURE 2:**
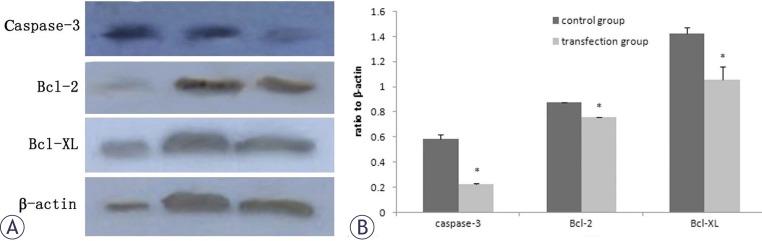
Protein expression of caspase-3, bcl-2 and bcl-xl after miR-548c-5p transfection. (A) Western blot assay demonstrated decreased OD of caspase-3, Bcl-2, and Bcl-XL in transfection group when compared with control group. From left to right: CD90^−^ HepG2 group; control group; transfection group; (B) Quantification of caspase-3, bcl-2, and bcl-xl after miR-548c-5p transfection normalized to β-actin. Protein expression of caspase-3, Bcl-2, and Bcl-XL decreased significantly in transfected cells (*P<0.05).

The western blot assay showed that protein expression of caspase-3, Bcl-2, and Bcl-XL decreased significantly in CD90^+^ cells transfected with miR-548c-5p when compared with untransfected CD90^+^ cells (Figures 2A, B).

### miR-548c-5p inhibits CD90^+^ cell proliferation

**FIGURE 3:**
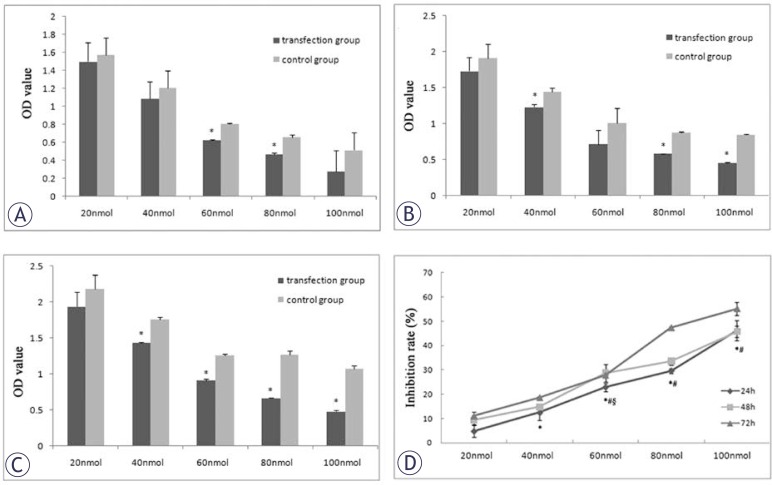
miR-548c-5p inhibits CD90^+^ cell proliferation. IR of CD90^+^ HepG2 cells increased markedly following transfection and the IR increased over time. (A) OD at 24 h after miR-548c-5p transfection. OD decreased at 24 h after miR-548c-5p transfection in MTT assay; (B) OD at 48 h after miR-548c-5p transfection. OD decreased at 48 h after miR-548c-5p transfection in MTT assay; (C) OD at 72 h after miR-548c-5p transfection. OD decreased at 72 h after miR-548c-5p transfection in MTT assay; (D) IR after transfection with miR-548c-5p mimics (MTT). IR of CD90^+^ HepG2 cells increased markedly following transfection with miR-548c-5p mimics and IR increased over time.* 72h verses 24h, P<0.05; # 72h verses 48h, P<0.05; 48h verses 24h, P<0.05

MTT assay was performed to examine the IR of CD90^+^ HepG2 cell proliferation after miR-548c-5p transfection. In miR-548c-5p transfected cells, the OD was decreased at 24 h, 48 h, and 72 h after the transfection (Figures 3A, B, C). IR of CD90^+^ HepG2 cells following the transfection increased markedly as compared to untransfected CD90^+^ HepG2 cells and the IR increased over time (Figures 3D).

### miR-548c-5p inhibits migration and invasion of CD90^+^ cells

**FIGURE 4:**
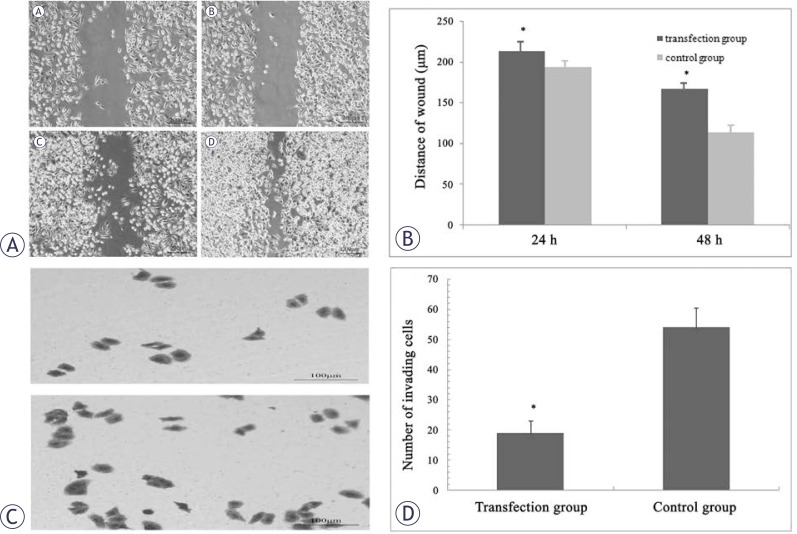
Wound healing assay (A, B) and transwell invasion assay (C, D). (A) Wound healing assay (1): miR-548c-5p transfection group (24 h); (2): control group (24 h); (3): miR-548c-5p transfection group (48 h); (4): control group (48 h); (B) Distance of wound was correlated to cell migration ability. Results showed that cell migration was inhibited in transfection group at 24 h and 48 h after miR-548c-5p transfection (*P*<0.05); (C) Cells penetrating the membrane in transwell invasion assay. The number of CD90^+^ HepG2 cells penetrating the membrane decreased at 48 h after miR-548c-5p transfection, upper: transfection group; lower: control group; (D) Changes in the number of cells penetrating the membrane in transwell invasion assay. The number of cells penetrating the membrane was lower in the transfection group than that in the control group. *P<0. 05 *vs* control group.

The wound healing assay showed that the migration ability of CD90^+^ HepG2 cells was significantly compromised at 48 h after miR-548c-5p transfection when compared with untransfected CD90^+^ HepG2 cells (Figures 4A, B).

The transwell invasion assay revealed that the number of CD90^+^ HepG2 cells penetrating the membrane decreased at 48 h after miR-548c-5p transfection as compared to the control group (Figures 4C). The number of cells penetrating the membrane was markedly lower in the transfected cells as compared to untransfected cells (P<0.05) (Figures 4D).

### Apoptosis of CD90^+^ cells following transfection with miR-548c-5p

**FIGURE 5:**
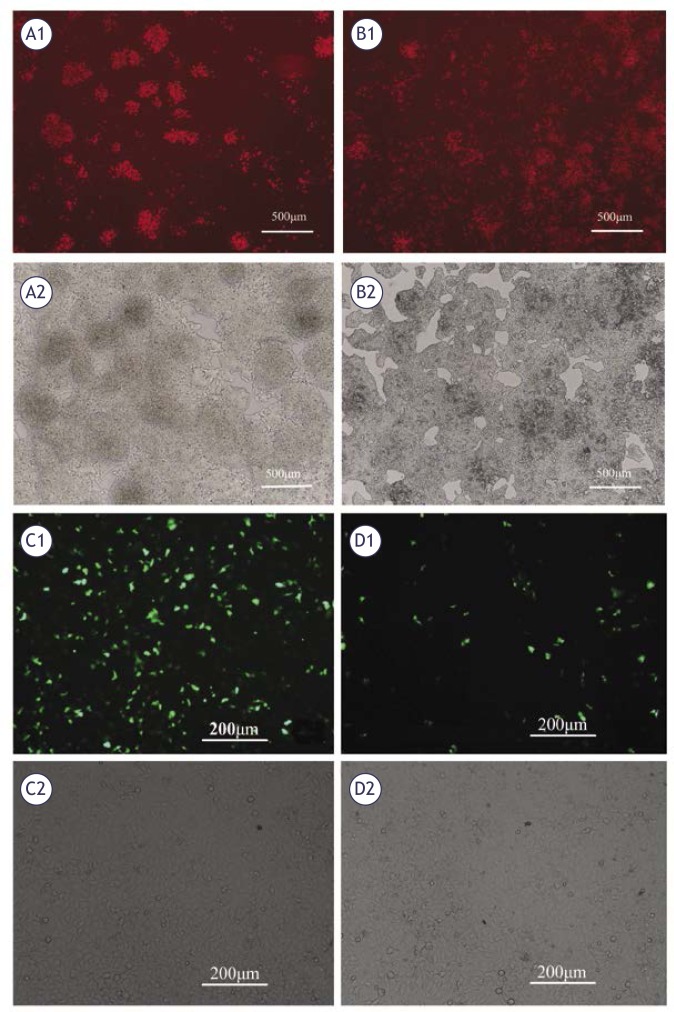
Apoptosis of CD90^+^ HepG2 cells after miR-548c-5p transfection. PI staining (A1, B1) showed apoptosis of CD90^+^ HepG2 cells increased in the transfection group at 48 h after miR-548c-5p transfection as compared to the control group. (A1) PI positive cells in the transfection group; (A2) cells under normal light in the transfection group; (B1) PI positive cells in the control group; (B2) cells under normal light in the control group. Annexin V-FITC staining (C1, D1) showed the apoptotic CD90^+^ HepG2 cells increased in the transfection group. (C1) Annexin V-FITC positive cells in the transfection group; (C2) cells under normal light in the transfection group; (D1) Annexin V-FITC positive cells in the control group; (D2) cells under normal light in the control group.

The apoptosis of CD90^+^ HepG2 cells was observed under a fluorescence microscope. Propidium iodide staining showed the increased number of late apoptotic CD90^+^ HepG2 cells at 48 h after miR-548c-5p transfection as compared to the un-transfected cells (Figures 5A1 and B1). Annexin V-FITC staining also revealed the increased number of apoptotic CD90^+^ HepG2 cells after the transfection (Figures 5C1 and D1).

## Discussion

Our study showed that miR-548c-5p could down-regulate the expression of *β-catenin, Tg737, bcl-2, bcl-XL*, and c*aspase-3*, inhibit the proliferation, migration, and invasion and promote apoptosis of CD90^+^ HepG2 cells. miR-548c-5p might be a suppressor of LCSC-like cells.

miR-548c-5p had a potential influence on apoptosis. miR-548c-5p may be a regulator of *caspase-3, bcl-2* and *bcl-XL* in the CD90^+^ HepG2 cells. In the present study, *caspase-3, bcl-2*, and *bcl-XL* were down-regulated in the CD90^+^ HepG2 cells after the transfection with miR-548c-5p mimics. The relationship between apoptosis and tumours is one of the focuses in recent years. The increase of anti-apoptosis is an important process in the development and progression of tumors.^18^ The caspase family is one of the key participants in the apoptosis.^20^ Caspase-3 is activated through either extrinsic or intrinsic signalling pathway and is a key effector caspase for proteolysis of other effector caspases and cellular proteins. Many apoptosis-triggering factors have a relationship with caspase-3.^21^,^22^ Vaishnav *et al*. reported JNK Interacting Protein-1(JIP1) to be a target for caspase-3 mediated cleavage in response to both chemical and receptor mediated apoptotic stimuli.^22^ Caspase-3 cleaves JIP1 at two sites leading to the disassembly of the JNK/JIP signalling complex thought to be required for JNK activation. Cleavage of both ERK and JNK scaffold proteins by caspases suggests a general mechanism for the regulation MAPK signalling during apoptotic cell death. In the present study, caspase-3 was down-regulated in the CD90^+^ HepG2 cells, which may influence the caspase-3 dependent signal transduction pathway. Although down-regulation of caspase-3 was inconsistent with the findings that the reduction of proliferation, we postulated that there might be other mechanisms related to caspase-3 affecting the apoptosis following the transfection.

The Bcl-2 family was first discovered among multiple genes of programmed cell death-regulating families. In recent years, the Bcl-2 family has aroused special attention.^23^ Bcl-2 and bcl-XL can inhibit the apoptosis. Kim *et al*. found that Bcl-2 was highly expressed in the gastrointestinal tumors.^24^ As apoptosis was inhibited, the cancer growth increased, resulting in poor prognosis and poor response to the treatment.^25^ We found in the present study that *bcl-2* and *bcl-XL* were down-regulated in the CD90^+^ HepG2 cells. It is well known that *bcl-2* is an anti-apototic gene, and the inhibitory effect of *bcl-2* on apoptosis may be exerted through regulating the intracellular signal transduction by preventing Ca^2+^ from entering cells.^26^ As bcl-2 can inhibit apoptosis, we postulate that miR-548c-5p may promote the apoptosis and inhibit the proliferation of CD90^+^ HepG2 cells partly through down-regulating bcl-2. It is predicted by TargetScan (http://www.targetscan.org), miRanda (http://www.microrna.org/microrna/home.do) and PicTar (http://pictar.bio.nyu.edu/) methods that *bcl-2* and *bcl-XL* may be possible downstream regulating genes of miR-548c-5p, but whether miRNA directly acts on the *bcl-2* and *bcl-XL*, or regulates them through other pathways is needed to be further confirmed in more studies. Our study showed that miR-548c-5p could promote apoptosis of CD90^+^ HepG2 cells and the *β-catenin* expression was decreased. It has been reported that β-catenin is an important mediator of the classical Wnt pathway and plays an important role in the tumour growth.^27^ Lee *et al*. reported that inhibitor of DNA binding 3 mediated X-ray-induced apoptosis of keratinocytes through down-regulation of endogenous β-catenin level in HaCaT keratinocytes.^28^ Selenite could induce apoptosis through reactive oxygen species (ROS)-dependent inhibition of AKT/b-catenin signaling pathway.^29^ We speculate that the increased apoptosis of CD90^+^ HepG2 cells may be attributed to the decreased β-catenin following miR-548c-5p transfection. Whether the increased apoptosis of CD90^+^ HepG2 cells following miR-548c-5p transfection is mediated by other pathways is required to be further investigated.

Apoptosis-related genes were differentially expressed in the CD90^+^ HepG2 cells, including down-regulation of *caspase-3* and *bcl-2*, and up-regulation of *caspase-8*, though *bcl-XL* and *p53* remained unchanged. This may attribute to the imbalance between apoptotic and anti-apoptotic processes in the CD90^+^ HepG2 cells, affecting the development and progression of CD90^+^ HepG2 cells. Nevertheless, apoptosis is an extremely complex process, and ultimately depends on various apoptosis-related genes.

miRNAs are new groups of carcinogenic genes or tumour suppressor genes, playing important roles in the carcinogenesis.^30^–^32^ Significant differences in the miRNA expression have been reported between liver cancer and para-liver cancer tissues.^33^,^34^ Some studies have shown that miR-200a is related to the β-catenin and tumour growth factor.^35^,^36^ Silencing of miR-200a gene could promote the occurrence of liver cancer.^36^ Our results showed the expression of miR-548c-5p, miR-145, miR-375 and miR-874 were decreased in the CD90^+^ HepG2 cells, while that of miR-155, miR-198 and miR-1289 increased. These changes indicate that CD90^+^ HepG2 cells may differ from CD90^−^ HepG2 cells in miRNAs expression, of which miR-548c-5p affects proliferation and invasion of CD90^+^ HepG2 cells demonstrated on our study. What roles other miRNAs play in the CD90^+^ HepG2 cells remain still unclear.

In summary, the *caspase-3* and *bcl-2* in the CD90^+^ HepG2 cells are down-regulated and *caspase-8* up-regulated, while *bcl-XL* remains unchanged. It is postulated that there is an imbalance between apoptosis and anti-apoptosis in the LCSC-like cells, resulting in the alteration of biological features of LCSC-like cells. Levels of several miRNAs are also changed in the CD90^+^ HepG2 cells, of which miR-548c-5p is found to affect several apoptosis-related genes including *bcl-2, bcl-XL* and *caspase-3*, indicating miR-548c-5p plays an important role in the apoptosis of LCSC-like cells. miR-548c-5p also affects the proliferation, migration, and invasion of CD90^+^ HepG2 cells and may thus act as a suppressor of LCSC-like cells. As a whole, miR-548c-5p plays a regulatory role in the primary liver cancer. Although increasing studies on cancer cells and LCSC-like cells have emerged, more efforts are needed to elucidate them in an in-depth way.
